# Efficacy of Acceptance and Commitment Therapy in Reducing Suicidal Ideation and Deliberate Self-Harm: Systematic Review

**DOI:** 10.2196/10732

**Published:** 2018-06-25

**Authors:** Joseph Tighe, Jennifer Nicholas, Fiona Shand, Helen Christensen

**Affiliations:** ^1^ Black Dog Institute School of Psychiatry University of New South Wales Sydney Australia; ^2^ Black Dog Institute University of New South Wales Sydney Australia

**Keywords:** suicidal ideation, suicide, deliberate self-harm, depression, mental health, acceptance and commitment therapy, cognitive behavioral therapy, mHealth, psychology, ACT

## Abstract

**Background:**

Since its emergence in the 1980s, acceptance and commitment therapy (ACT) has become a reputable evidence-based psychological therapy for certain disorders. Trials examining the efficacy of ACT are spread across a broad spectrum of presentations, such as chronic pain, anxiety, and depression. Nevertheless, ACT has very rarely been trialed as an intervention for suicidal ideation (SI) or deliberate self-harm (DSH).

**Objective:**

The objective of this review is to assess the efficacy of ACT in reducing SI and DSH and to examine the suitability of reported SI, DSH, and other measures in determining the efficacy of ACT.

**Methods:**

We systematically reviewed studies on ACT as intervention for SI and self-harm. Electronic databases, including MEDLINE, PubMed, EMBASE, PsycINFO, SCOPUS, Cochrane Central Register of Controlled Trials, and the Cochrane Database of Systematic Reviews, were searched. The reference lists of included studies and relevant systematic reviews were examined to identify additional publications. Search terms were identified with reference to the terminology used in previous review papers on ACT and suicide prevention. The study design was not restricted to randomized controlled trials. Screening was completed by 2 reviewers, and all duplicates were removed. Publications were excluded if they were not published in English, were multicomponent therapy or were not based on ACT, or lacked a validated measure or structured reporting of SI/DSH outcomes.

**Results:**

After removing the duplicates, 554 articles were screened for relevance. Following the screening, 5 studies that used ACT as an intervention for suicidal or self-harming individuals were identified. The studies used diverse methodologies and included 2 case studies, 2 pre–post studies, and 1 mHealth randomized controlled trial.

**Conclusions:**

The review found that ACT is effective in reducing SI in the 2 pre–post studies but not in other studies. However, given the small number and lack of methodological rigor of the studies included in this review, insufficient evidence exists for the recommendation of ACT as an intervention for SI or DSH.

## Introduction

Suicide is one of the leading causes of death worldwide, and the World Health Organization attributes over 800,000 deaths per year to suicide [1]. It has tragic and drastic effects, and, globally, for every person who dies by suicide, 20 more people attempt to take their life but do not die [1]. Many are bereaved in the aftermath of a suicide. Family members, friends, and those close to the deceased are at an increased risk of suicide themselves. Therefore, understanding and implementing methods for reducing suicide are critical, as is the prioritization and recognition of suicide as a solvable major public health problem.

For the purpose of this review, suicide is defined as the act of deliberately killing oneself, and deliberate self-harm (DSH) is defined as any nonfatal suicidal behavior, such as intentional self-injury, poisoning, or self-harm with or without a fatal intent. Suicide and DSH are preventable, and therapeutic approaches that specifically target suicidal ideation (SI) provide successful results [2-4]. Cognitive behavioral therapy (CBT) can reduce SI and treat depression and insomnia [5]. The use of CBT as an intervention for suicidality has been tested in a number of randomized controlled trials (RCTs), with some evidence for its efficacy [6-8]. CBT for suicide prevention and CBT for suicidal patients have also had positive effects on SI [2,3].

An evidence base for the “third wave” of CBTs, such as dialectical behavioral therapy (DBT), mindfulness-based CBT (MBCT), and acceptance and commitment therapy (ACT), has been established over the last 15 years [9]. DBT is highly effective in treating presentations of self-harm among those with borderline personality disorder and is frequently delivered as group therapy [10]. MBCT has become an acceptable alternative to CBT. In MBCT, the practice of mindfulness activities is considered a useful addition to standard CBT activities [11]. Given the strong evidence for the efficacy of third-wave therapies for other indications [9-12], it is worth examining whether ACT shows promise in the area of suicide prevention.

ACT attempts to increase psychological flexibility mainly by targeting experiential avoidance—the tendency to avoid unwanted thoughts or emotions [13]. The 6 core processes of ACT are as follows: (1) acceptance of uncomfortable private experiences (thoughts, feelings, or physical sensations); (2) cognitive defusion/distancing from one’s own uncomfortable thoughts; (3) being present (directing attention to present events and experiences rather than focusing on the past or future); (4) self-awareness in the present moment through the “observing self;” (5) identification of personal values; and (6) commitment to action in line with the identified values. Since the publication of *Acceptance and Commitment Therapy* in 1999 by the treatment’s cocreators—Steven Hayes, Kirk Strosahl, and Kelly Wilson [13]—the number of ACT-based RCTs has increased [12]. Unsurprisingly, ACT has been used to treat common mental health conditions, such as depression, anxiety, addiction, and stress, as well as physical conditions, such as chronic pain [12,14,15]. However, trials that examine the efficacy of ACT in targeting SI/DSH continue to lack.

There is good reason to hypothesize that ACT may be effective in reducing SI and DSH by improving psychological flexibility [16]. Some of the predominant psychological frameworks that attempt to explain suicide, particularly the entrapment/cry of pain model, include escape from pain as a key factor [17-19]. Escape, referred to as experiential avoidance, is one of the key target areas of ACT treatment. The application of mindfulness skills, acceptance of distress, and defusion from distressing thoughts may improve an individual’s ability to live with the discomfort of severe emotional pain. Finally, the identification of personal values and taking up of positive action aligned with these values may lead to an integrated individual, thereby improving wellbeing.

To date, reviews of ACT for SI or DSH have not been published. A 2014 meta-analysis of the efficacy of ACT examined 60 RCTs that focused on psychiatric disorders, somatic disorders, and stress at work [12]. Of these studies, none examined SI or DSH. A 2016 meta-analyses and metaregression of studies that examined the effectiveness of psychotherapy in reducing suicidal attempts and nonsuicidal self-injury rates included 32 RCTs, none of which included ACT as a treatment despite the brief mention of its promise [20]. Hacker et al [21] highlighted the efficacy of ACT in systematic reviews but were critical of the broad range of presentations reviewed and the lack of specificity on common mental health problems. We have attempted to overcome this limitation with our focus. Given that some authors have proposed that ACT treatment has the potential to be an effective intervention for suicidal individuals [22,23], the available evidence must be reviewed. For this review, we aimed to examine (1) whether ACT is an effective treatment for SI or DSH and (2) the suitability of reported SI, DSH, and other measures in determining the efficacy of ACT.

## Methods

This review was conducted in accordance with the Preferred Reporting Items for Systematic Reviews and Meta-analyses guidelines [24].

### Search Strategy and Selection Criteria

The following electronic databases were systematically searched: MEDLINE, PubMed, EMBASE, PsycINFO, SCOPUS, Cochrane Central Register of Controlled Trials, and the Cochrane Database of Systematic Reviews. A comprehensive set of search terms was identified with reference to terminology used in previous review papers for ACT [9,12] and suicide prevention [4,25]; these terms were combined with MeSH terms relevant to each of the databases. Search terms included “acceptance and commitment therapy” or “acceptance-based therapy” and either “suicide,” “ideation,” “assisted suicide*,” “attempted suicid*,” “self-injurious behavior,” “self-mutilation,” “self-harm,” “self-poison*,” “self-inflicted wounds,” “drug overdose,” “overdose,” or “parasuicid*.” In addition, the reference lists of all included studies and relevant reviews were examined to identify additional relevant publications.

Despite the emergence of ACT in the late 1990s, no date restrictions were placed on searches that were completed on December 11, 2017. A study was eligible for inclusion if it satisfied the following criteria: (1) The study used ACT as an intervention. Multicomponent therapy types were excluded because of our interest in examining the efficacy of ACT when used as a standalone therapeutic intervention. Interventions could be delivered to individuals or groups or through technology. (2) The study assessed suicidal behavior by using a validated measure or structured reporting. Suicidal behavior was defined in its broadest terms and ranged from SI to the various forms of self-harm indicated in the search terms. (3) The study is an original peer-reviewed article published in English. Given the recency of third-wave interventions, study design was not restricted to RCTs. Instead, all research designs were included (eg RCTs, quasiexperimental, pre–post, single group, and case studies). Finally, the age of participants was unrestricted. Appendix 1 provides search term details.

### Selection Process

After the removal of duplicates, 2 researchers (JT and JN) independently reviewed the relevance of all titles and abstracts that were returned by the search. Studies considered irrelevant by both the reviewers were excluded. The full texts of the remaining articles were then independently examined by the same 2 authors to confirm eligibility. Included articles and reasons for exclusion were compared to achieve consensus and, where necessary, disputes were settled by a third researcher (FS).

### Data Extraction

One author (JT) extracted the study characteristics and outcome variables, which were independently checked by JN. The following variables were extracted: author name, publication year, sample type, control group details, program format, participant age, program length, and follow-up interval. Outcome data on SI/DSH, depression, and psychological flexibility (acceptance and mindfulness) were also extracted.

### Risk of Bias

The study quality and risk of bias of the included RCTs were assessed using the Cochrane Collaboration “Risk of Bias” tool [26].

## Results

### Study Selection

**Figure 1:**
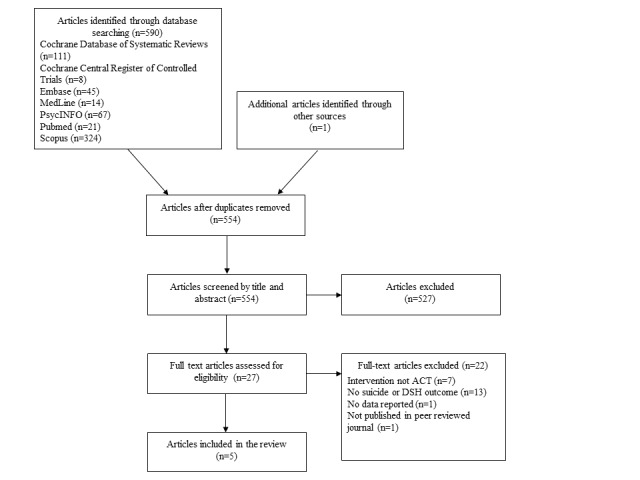
Study selection flow diagram. ACT: acceptance and commitment therapy; DSH: deliberate self-harm.

The database search identified 590 articles and 1 article was found through a google scholar search. After removing duplicates (n=37), the titles and abstracts of the remaining 554 articles were screened for relevance, and 527 articles were excluded. The full texts of the 27 remaining articles were then examined, and 5 studies were finally included in the review (Figure 1).

### Study Characteristics

The characteristics of the included studies are presented in Table 1, and the outcome variables are presented in Table 2. All 5 studies focused on individual therapy rather than group therapy. This review included 1 mHealth RCT (N=61), 2 pre–post studies (N=981, N=35), and 2 case studies (N=2, N=3).

### Study Quality and Risk of Bias

The risk of bias was evaluated for the 1 RCT study included in this review, as shown below (Table 3). Overall, the study was judged to have a low risk of bias across all domains, except for the blinding of participants and personnel (high risk).

### Included Studies

#### Study 1: Tighe et al (2017)

This study [27] aimed to evaluate the effectiveness of an ACT-based self-help mobile app (ibobbly) that targets SI, depression, psychological distress, and impulsivity among Aboriginal and Torres Strait Islander youth in remote Australia. A two-arm randomized controlled trial comprising 61 Aboriginal and Torres Strait Islander Australians aged 18­-35 years was conducted in the Kimberley region of Western Australia. The mean age of the participants was 26 years (SD 8.1). Among the participants, 64% were identified as female. Participants in group 1 immediately received the ibobbly app, which delivered acceptance-based therapy over 6 weeks. Participants in group 2 were waitlisted for 6 weeks and then received the app for the following 6 weeks. The primary outcome was the frequency and intensity of SI in the previous weeks as identified through the Depressive Symptom Inventory—Suicidality Subscale (DSI-SS) [28]. Secondary outcomes were the Patient Health Questionnaire 9 (PHQ-9) [29], the Kessler Psychological Distress Scale (K10) [30], and the Barratt Impulsivity Scale [31]. The key outcome variable for this review was the DSI-SS.

Although a significant improvement in suicidality was reported postintervention in the ibobbly arm (*t*=2.40; df=58.1; *P*=.02), this difference was not significant compared with that reported in the waitlist arm (*t*=1.05; df=57.8; *P*=.30). However, participants in the ibobbly group showed substantial and statistically significant reductions in PHQ-9 and K10 scores of 42% and 28%, respectively, compared with those in the waitlist group (*P*=.007 and *P*=.02, respectively). No differences were observed in impulsivity. The SI, distress, and depression measures of the waitlisted participants improved after 6 weeks of using the app.

**Table 1:** Summary of included studies.

Authors	Sample	Study intervention	Control condition	Suicide/Self-harm specific outcome variables	Length of intervention	Follow-up intervals	SI^a^/DSH^b^ results
Tighe et al (2017)	Aboriginal and Torres Strait Islander Australian youth ages 18-35 (N=61)	ACT^c^ mHealth app (ibobbly)	Waitlist (6 weeks)	SI (DSI-SS^d^)	Self-help app over 6 weeks	None	30% reduction in SI but nonsignificant
Walser et al (2015)	Veterans (N=981)	ACT-D^e^ (specifically designed for veterans)	None	SI (BDI-II^f^, 1 SI Item)	12-16 psychotherapy sessions	None	20.5% reduction in prevalence of SI among participants
Ducasse et al (2014)	Psychiatric patients (N=37)	ACT	None	SI (C-SSRS^g^), Scale for Suicidal Ideation (SSI score) and suicidal ideation on a visual analog scale (self-report)	7 weekly 2 h sessions	1 week and 3 months	Significant reductions in all SI measures at 1-week and 3-month follow-up
Luoma & Valatte (2012)	Case studies (N=2)	ACT	None	Case study reports on suicidal ideation and self-harm.	38 psychotherapy sessions (n=1)	1 year (n=1) and unspecified (n=1)	Reductions in SI (N=2).
Rassaque et al (2012)	Case studies (N=3)	ACT	None	Interviews and hospital ward reports measuring suicidal ideation and self-harm expression	20 minute one-to-one sessions over 2 to 3 weeks	Unspecified	“a marked reduction in self-harm and suicidal ideation” (n=1), “changes in expression of self-harm or suicidal ideation” (n=2)

^a^SI: suicidal ideation.

^b^DSH: deliberate self-harm.

^c^ACT: acceptance and commitment therapy.

^d^DSI-SS: Depressive Symptom Inventory—Suicidality Subscale.

^e^ACT-D: acceptance and commitment therapy for depression.

^f^BDI-II: Beck Depressive Inventory.

^g^C-SSRS: Columbia-Suicide Severity Rating Scale.

**Table 2:** Outcome measures reported in the included studies. An "X" indicates the presence of the measure.

Measure	Tighe et al (2017)	Walser et al (2015)	Ducasse et al (2014)	Luoma and Valatte (2012)	Razzaque et al (2012)
Scale for Suicidal Ideation			X		
Suicidal Ideation (Self-Assessment Visual Analog scale)			X		
Columbia-Suicide Severity Rating Scale (suicidal ideation subscore=severity and intensity items)			X		
Suicidal Ideation	X				
Beck Depressive Inventory-II		X	X		
Patient Health Questionnaire	X				
Kessler 10	X				
Barrett Impulsivity Scale	X				
Acceptance and Action Questionnaire		X	X		
Five-Facet Mindfulness Questionnaire		X		X (n=1)	
Mini International Neuropsychiatric Interview (French version)			X		
Screening Interview for Axis II Disorder			X		
Inventory of Depressive Symptomatology			X		
Functioning Assessment Short Test			X		
Pharmacological treatment and number of visits for psychiatric emergencies (previous 3 months)			X		
Psychological pain on a visual analog scale			X		
State-Trait Anxiety Inventory			X		
Beck Hopelessness Scale			X		
World Health Organization Quality of Life measure			X		
Clinical Global Index					X

**Table 3:** Risk of bias for the randomized controlled study reported by Tighe et al (2017) [27].

Entry	Judgment	Support for judgment
Random sequence generation (selection bias)	Low risk	Quote: “using block randomization stratified by gender (16 per block), using computer-generated randomization” Comment: Probably done.
Allocation concealment (selection bias)	Low risk	Quote: “Each block randomization was performed offline by a member of the research team at the Black Dog Institute and sent to the research officer in Broome.” Comment: Probably done.
Blinding of participants and personnel (performance bias)	High risk	Quote: “research officer in Broome who was responsible for and not blind to the intervention allocation” Comment: Probably not done.
Blinding of outcome assessment (detection bias; patient-reported outcomes)	Low risk	No blinding of outcome assessment used. Outcome measures were self-reported and it is unlikely that the outcome measurement would be influenced by blinding.
Incomplete outcome data addressed (attrition bias)	Low risk	Follow-up: minimal missing data. 2/31 missing from intervention group; 0/30 missing from control group. Reasons unlikely to be related to outcome.
Selective reporting (reporting bias)	Low risk	Quote: “The study protocol has been published.”
		

#### Study 2: Walser et al (2015)

Walser et al (2015) [32] conducted a pre–post evaluation to measure the effectiveness of ACT in treating depression and SI in a group of veterans (N=981). They utilized a modified version of ACT for depression (ACT-D) specifically designed for veterans. The mean age of the participants was 50.5 years (SD 12.5), and 75% of the participants were identified as male. The intervention, which encompassed the 6 core processes of ACT, was administered over 12-16 individual psychotherapy sessions. Depression and SI were measured using the Beck Depressive Inventory (BDI-II) [33]. Experiential acceptance and mindfulness (2 goals of ACT) were also measured to determine their effect on depression and SI. Experiential acceptance was measured with the Acceptance and Action Questionnaire [34], and mindfulness was measured with the Five-Facet Mindfulness Questionnaire (FFMQ) [35]. The key outcome variable for this review is the BDI-II (suicide item).

The percentage of participants with no SI increased from 44% at baseline to 65% at follow-up because SI scores significantly decreased. Depression significantly reduced, as indicated by BDI-II scores. Specifically, scores decreased by 32% and 40% in participants with and without SI at baseline, respectively. Increases in mindfulness scores were associated with a reduction in depression severity across time (*P*=.04). Decreases in experiential avoidance scores were associated with a reduction in SI across time (*P*=.02). However, mindfulness scores were not significantly related to SI scores over time.

#### Study 3: Ducasse et al (2014)

In 2014, Ducasse et al [36] conducted a pilot study on adjunctive ACT with a cohort of French outpatients (N=35) who were diagnosed with suicidal behavior disorder on the basis of DSM-5 criteria (Section III) [37]. All had attempted suicide in the previous year. The study featured an ACT program as an add-on to treatment-as-usual. Among the participants, 57% were identified as male. The median age was 38.4 years (18-60, no mean age reported). The intervention was delivered through 7 individual weekly sessions. Each session lasted for 2 h, and written summaries were provided at the end of each session for practice at home. One suicide was reported in the first month of the study. The authors reported that this suicide had no clear link to the study. Measures were taken at 1 week prior to program, 1 week postprogram, and 3 months after program completion.

The Columbia-Suicide Severity Rating Scale (C-SSRS) [38], Scale for Suicidal Ideation (SSI) [39], and the Inventory of Depressive Symptomatology (IDS-C30) [40] were administered. Self-assessments included the following: (1) SI on a visual analog scale from 0 (none) to 10 (maximum); (2) depression severity using the Beck Depression Inventory-II [33]; (3) psychological pain on a visual analog scale from 0 (none) to 10 (maximum); (4) anxiety state using the State-Trait Anxiety Inventory [41]; (5) hopelessness using the Beck Hopelessness Scale [42]; (6) quality of life using the World Health Organization Quality of Life measure [43]; and (7) acceptance using the Acceptance and Action Questionnaire (AAQ) [34]. The key outcome variables for this review were the C-SSRS and the SSI.

All scores between 3 visits significantly decreased (*P*<.001). The C-SSRS SI subscore and SSI score (7, 0-22 vs 0, 0-10; *P*<.001) significantly decreased from preintervention to the 1-week postprogram follow-up (20, 0-30 vs 0, 0-20, respectively; *P*<.001). The intensity of current and previous SI during the last 15 days significantly decreased between inclusion and 1-week postprogram follow-up (1, 0-10 vs 0, 0-3; 2, 0-9 vs 0 ,0-5, respectively; both *P*<.001). The SI (C-SSRS) subscore was correlated to the AAQ score (*P*=.04, r=−0.37) but not to BDI-II score. At 3-month follow-up, all SI scores remained significantly low. However, the actual scores were not reported. This study also reported a reduction in depression between baseline and 1-week follow-up (BDI-II [13, 2-28 vs 4.5, 0-24; *P*<.001]) and IDS-C30 scores (28, 12-61 vs 8, 0-31; *P*<.001).

#### Study 4: Luoma and Villatte (2012)

Luoma and Villatte [44] reported on 2 case studies. One case included 1 patient with chronic SI, and the other included 1 patient with transient SI. The patients were treated “largely from an ACT perspective.”

##### Case Study 1

Anne (22) underwent ACT therapy while on a waitlist for DBT for assistance with intense emotional dysregulation, deliberate self-injury, and suicide risk. Anne had a history of suicide attempts, had been struggling with persistent suicidal thoughts, and met the criteria for multiple Axis I and II disorders. Over the course of therapy, significant increases on the FFMQ [35] were reported in line with overall decreases in symptomatology and borderline features. The authors reported a reduction in SI and DSH but did not state the measures used. After 18 treatment sessions, Anne’s level of psychological distress had fallen to subclinical levels on 2 symptom inventories (not specified in the paper). Treatment continued biweekly for 20 additional weeks before termination. At 1-year follow-up, Anne’s mindfulness scores remained high, and she no longer met the current criteria for any psychological disorder.

##### Case Study 2

Considerably limited background information was provided for the second case study, which featured a 47-year-old male. Mark initiated therapy after attempting suicide shortly after losing his family in a motor vehicle accident. The authors reported that after 6 months of ACT, Mark no longer considered suicide as a viable option. However, the authors did not describe the psychological measures used in the case.

#### Study 5: Razzaque (2012)

Razzaque’s study [45] detailed the delivery of ACT to 3 patients in the psychiatric intensive care unit of Goodmayes Hospital in East London. All 3 had a lengthy history of regular bouts of violence toward themselves or others. The first had a primary diagnosis of schizoaffective disorder, with an average of 2-3 psychiatric admissions per year. The second and third participants were diagnosed with bipolar affective disorder. Their presentations included frequent bouts of violence toward themselves, family members, carers, and ward staff. The treatment consisted of 20 min one-to-one ACT sessions delivered daily over 2-3 weeks.

Violence and aggression toward others were measured in addition to self-harm expression and/or SI. Aggressive and abusive behaviors were recorded in regular nursing shift reports. No specific measure was used for the measurement of self-harm or SI. However, interviews and ward reviews were used to record changes in self-harm expression and SI. In addition to reductions in derogatory auditory hallucinations, self-harm and SI markedly reduced for the patient diagnosed with schizoaffective disorder. The aggressive and abusive behaviors of the 2 patients diagnosed with bipolar disorder reduced.

## Discussion

### Principal Findings

This review aimed to understand if ACT can successfully reduce SI or self-harm and to examine the suitability of the measures used in the included studies. The review found few empirical investigations on ACT that specifically target the reduction of SI/DSH, with only 1 RCT among the 5 studies. This is the first review we are aware of that has examined the impact of ACT on the reduction of SI/DSH.

All 5 studies examined SI by using various measures, and only the 2 case studies examined DSH. All studies reported a reduction in SI, and both case studies reported reductions in DSH over the course of the interventions. The degree of the reduction in SI varied: Tighe et al [27] reported a nonsignficant 30% reduction in SI scores, and Walser et al reported a significant 20.5% reduction in the prevalence of SI among participants at follow-up [32]. Ducasse et al [36] reported significant reductions in all SI measures at 1-week and 3-month follow ups [36]. However, the effective evaluation of ACT treatment was diminished given that the ACT component of the participants’ therapy was provided as an add-on to treatment-as-usual. The authors of this trial concluded that ACT might help reduce the intensity and frequency of SI by increasing acceptance and valued action and by reducing risk factors, such as hopelessness and psychological pain [36]. The 2 case studies measured and reported a reduction in SI and DSH [44,45].

Although this review focuses on SI and DSH, the secondary measures of depression, acceptance, and mindfulness are crucial for evaluating the effectiveness of ACT. Among the 5 studies included in this review, 3 reported outcomes for depression. This review supports the existing body of evidence that highlights the effectiveness of ACT in this common presentation [12]. Tighe et al [27] found that depression scores in the intervention group significantly reduced by 42% relative to that in the waitlist group. Walser et al [32] reported a 32% and 40% reduction in the depression scores of the cohort of veterans with and without recorded SI at baseline, respectively. The pre–post study conducted by Ducasse et al [36] lacked a control group but showed significant reductions for all measures, including depression. The positive results of ACT on depression support recent research that highlights the potential of ACT as an intervention for common mental health conditions, such as depression and anxiety [12,46]. Furthermore, the reductions in depression and SI reported by the included studies support previous research demonstrating that reductions in depression can lead to reductions in SI [47,48]. The use of the BDI-II by Walser et al [32] to measure depression and the presence of SI provided limited data on SI/DSH. The use of focused SI/DSH measures in future studies would likely enrich analyses. Notably, most measures were not common between studies. However, 2 of the 5 studies included the AAQ and the FFMQ, which are key measures of acceptance and mindfulness (key goals of ACT treatment). Including these measures in future studies on ACT would be useful because their analyses could show potential associations with SI/DSH. The use of established standardized measures of SI/DSH in RCTs and case studies would also improve the evidence base for ACT.

The 5 studies included 3 distinct cohorts of participants. The mHealth RCT by Tighe et al [27] targeted Aboriginal and Torres Strait Islander youth. Walser et al [32] targeted veterans of the United States Army, and Ducasse et al [36] targeted French outpatients with a recent suicide attempt. In the 2 included case studies, 3 of the 5 participants were suffering from severe distress in hospital settings. Across these diverse populations, ACT was positively associated with a reduction in SI. The methodological quality of the studies was low, with just 1 (Tighe et al) [27] including a control group. The 2 studies that reported positive results for SI through case studies (N=2 and N=3) had limited participants and lacked robust research frameworks from which to draw conclusions [44,45]. Both studies report impressive reductions in SI for all the 5 individuals studied; however, the studies had numerous limitations, including lack of specificity in the measurement of SI and self-harm reports (apart from hospital ward reports). Details regarding treatment-as-usual, such as medication administration to participants, were not provided. Notably, the only RCT included in this review delivered therapy through a self-help mobile app [27]. Whether this is indicative of an increasing adoption of mHealth is worth considering [49-51].

All 5 studies showed that ACT is associated with symptom changes. This result provides a rationale for the largescale systematic evaluation of the efficacy of ACT as an intervention for suicidal behavior. Tighe et al [27] reported significant reductions in depression and distress on standardized measures and a 30% reduction in SI scores. This reduction, however, was nonsignificant between groups. All 5 participants whose case studies were presented improved significantly with marked reductions in SI/DSH [44,45]. In addition to the significant results for SI, the 2 pre–post studies showed improvements on the AAQ, a measure of psychological flexibility [32,36]. Walser et al [32] showed that their improvements on the AAQ are associated with a reduction in SI scores across time. Similarly, Ducasse et al [36] found a significant correlation between SI and AAQ scores, such that increased acceptance is associated with reduced SI. Accepting reality and reducing avoidance are fundamental aims of ACT, and this therapeutic work is aligned with the theories of suicide that focus on an individual’s sense of entrapment and/or desire to escape their current reality [17-19]. Further trials are needed to test how increases in experiential acceptance and mindfulness affect SI/DSH scores and whether targeting these factors might increase reductions in SI/DSH.

### Conclusion

The number of studies included in this review is too small to support the claim that ACT can effectively assist in the reduction of SI/DSH. Given the limited research that has been conducted on this topic to date, the efficacy of ACT in reducing SI or DSH requires further testing, particularly through controlled trials. The early evidence presented in this review suggests that the potential mechanisms of action, such as changes in experiential avoidance and mindfulness, should receive focus.

## Appendix

Multimedia Appendix 1 Database search terms.

## Abbreviations

ACT: acceptance and commitment therapy
ACT-D: acceptance and commitment therapy for depression
BDI-II: Beck Depressive Inventory
C-SSRS: Columbia-Suicide Severity Rating Scale
CBT: cognitive behavioral therapy
DBT: dialectical behavioral therapy
DSH: deliberate self-harm
DSI-SS: Depressive Symptom Inventory—Suicidality Subscale
FFMQ: Five-Facet Mindfulness Questionnaire
IDS-C30: Inventory of Depressive Symptomatology
K10: Kessler Psychological Distress Scale
MBCT: Mindfulness-Based Cognitive Therapy
PHQ-9: Patient Health Questionnaire 9
RCT: randomized controlled trial
SBD: Suicidal Behavior Disorder
SI: suicidal ideation
SSI: Scale for Suicidal Ideation
