# Supplementary material for: Efficacy of Acceptance and Commitment Therapy in Reducing Suicidal Ideation and Deliberate Self-Harm: Systematic Review
Source: JMIR Ment Health. 2018 Jun 25;5(2):e10732. doi: 10.2196/10732 (PMC6037942; doi:10.2196/10732)
Supplement: Multimedia Appendix 1 [file mental_v5i2e10732_app1.pdf]

# Appendix 1

**Database Search Terms**

**MEDLINE:**

1. suicide/ or suicidal ideation/ or suicide, assisted/ or suicide, attempted/
2. suicid*.mp.
3. self-injurious behavior/ or self mutilation/
4. self-harm*.mp.
5. self harm.mp.
6. self-poison*.mp.
7. self-inflicted wounds.mp.
8. Drug Overdose/ or overdose.mp.
9. parasuicid*.mp.
10. 1 or 2 or 3 or 4 or 5 or 6 or 7 or 8 or 9
11. "acceptance and commitment therapy".mp. or "Acceptance and Commitment Therapy"/
12. (acceptance-based adj4 therapy).mp. [mp=title, abstract, original title, name of substance word, subject heading word, keyword heading word, protocol supplementary concept word, rare disease supplementary concept word, unique identifier]
13. (acceptance adj6 therap*).mp. [mp=title, abstract, original title, name of substance word, subject heading word, keyword heading word, protocol supplementary concept word, rare disease supplementary concept word, unique identifier]
14. 11 or 12 or 13
15. 10 and 14

**PUBMED search terms:**

(((((acceptance and commitment and therapy))) OR (("acceptance and commitment therapy" or "Acceptance and Commitment Therapy"))) OR acceptance-based therapy) AND ((((((((((suicide/ OR suicidal ideation/ OR suicide, assisted/ OR suicide, attempted/)) OR suicid*) OR (self injurious behavior/ OR self mutilation)) OR self-harm*) OR self harm) OR self-poison*) OR self-inflicted wounds) OR (drug overdose OR overdose)) OR parasuicid*))

**SCOPUS search terms:**

( ( ( ( ( *acceptance* AND *commitment* AND *therapy* ) ) ) OR ( ( *"acceptance and commitment therapy"* OR *"Acceptance and Commitment Therapy"* ) ) ) OR *acceptance-based* AND *therapy* ) AND ( ( ( ( ( ( ( ( ( ( *suicide/* OR *suicidal* AND *ideation/* OR *suicide,* AND *assisted/* OR *suicide,* AND *attempted/* ) ) OR *suicid** ) OR ( *self* AND *injurious* AND *behavior/* OR *self* AND *mutilation* ) ) OR *self-harm** ) OR *self*

AND *harm* ) OR *self-poison** ) OR *self-inflicted* AND *wounds* ) OR ( *drug* AND *overdose* OR *overdose* ) ) OR *parasuicid** ) )
